# Protocol for a Study of Home-Based Hair Sample Collection to Monitor Antiretroviral Adherence Among a Multicultural Population of Women with HIV

**DOI:** 10.21203/rs.3.rs-10541677/v1

**Published:** 2026-09-14

**Authors:** Michelle Huang, Claudia M. Bonilla, Elisheva Knopf, Rachel Mpanu Mpanu, Lunthita M. Duthely

**Affiliations:** University of Miami Miller School of Medicine; Nova Southeastern University; Florida Atlantic University; University of Miami Miller School of Medicine; University of Miami Miller School of Medicine

**Keywords:** HIV, antiretroviral therapy adherence, hair sample analysis, home-based specimen collection, women with HIV, drug-level monitoring

## Abstract

**Background:**

Current blood-based antiretroviral (ARV) adherence measures reflect only short-term drug exposure, limiting their utility for assessing longitudinal adherence. Hair-based ARV concentration measurement offers an extended alternative but has not been adapted for multilingual populations or women at risk of HIV care disengagement.

**Objective:**

To evaluate the feasibility and acceptability of home-based hair sample collection for ARV adherence monitoring among a racially, ethnically, and linguistically diverse cohort of women with HIV (WHIV) in South Florida.

**Methods:**

This prospective mixed-methods study was conducted in three phases: theater testing of study procedures, hair sample collection, and qualitative data collection. Participants were WHIV enrolled from a Women's HIV Service at an academic medical center. Study consent and materials were delivered in English, Spanish, and Haitian Creole. Following an educational session on hair collection techniques, participants self-collected hair samples at home and mailed the samples to a laboratory for ARV concentration analysis. Feasibility and acceptability were further assessed through focus groups and individual interviews.

**Results:**

Of 40 participants recruited, 37 consented and 34 hair samples have been received and submitted for ARV concentration analysis. A total of 24 participants completed qualitative data collection via focus groups (n = 8) and individual in-depth interviews (n = 16). Full quantitative and qualitative analyses are ongoing.

**Conclusion:**

Preliminary findings suggest that home-based hair sample collection is feasible and acceptable among diverse WHIV. Culturally sensitive communication and participant-directed self-collection appear to facilitate engagement. Final results will inform the integration of hair-based adherence monitoring into HIV care for underrepresented populations.

## INTRODUCTION

HIV transmission remains a public health concern in the United States, with persistent regional and sub-population disparities [1]. This gap is especially pronounced in South Florida, where linguistically and culturally diverse women, including Haitian Creole- and Spanish-speaking communities, remain underrepresented in research and face compounded barriers to care [2-4]. Because sustained viral suppression is key to preventing vertical transmission, antiretroviral (ARV) adherence monitoring in women is particularly pertinent [5-7]. However, current blood-based ARV concentration measurements only capture drug levels up to the preceding 30 days [8], making this method vulnerable to the "white coat effect" (temporarily improved adherence ahead of clinical visits) and limiting its ability to assess longitudinal adherence [9].

Hair analysis has become an increasingly common non-invasive biomarker across medicine, offering a method for monitoring long-term ARV therapy [10]. Hair concentrations for ARVs like Tenofovir correlate strongly with dried blood spot standards (r = 0.76), reflect months of cumulative drug exposure, and have been more strongly associated with viral suppression than self-reported adherence [11, 12]. This longitudinal metric may help researchers more accurately identify patients at risk for treatment disengagement and adapt interventions accordingly.

Home-based ARV hair sampling, in which participants self-collect and mail their own hair, may increase convenience, privacy, and reduce participant burden. A prior study confirmed the feasibility and acceptability of home-based, small hair samples for measuring ARV adherence among a general, US-based population [13]. However, a subsequent longitudinal study was only conducted in English, with the study population including predominantly White (61%) men who have sex with men (MSM) (84%) [14]. Despite the disproportionate impact of adherence challenges on Black and Latina women in urban settings like South Florida, home-based hair sampling has not yet been adapted for multilingual populations or tailored to women; this study may address such gaps [15].

## Objectives

This exploratory, mixed-methods protocol aimed to adapt home-based, small-sample hair collection as a tool to assess ARV adherence among a racially, ethnically, and linguistically diverse, clinic-based cohort of women in South Florida who are at risk of disengagement from HIV care.

This protocol employed a sequential explanatory design, collecting quantitative data, followed by qualitative data, to inform the adaptation of the hair sampling method. Quantitative data allowed for participant characterization and examination of associations between known barriers and facilitators to HIV care adherence (e.g., depression, chronic stress, internalized HIV stigma, and resilience). ARV adherence was further assessed through laboratory analysis of self-collected hair samples. Qualitative methods supported the refinement of the hair sampling method, ensuring its feasibility and acceptability for future research with this population [16].

## Study Design

This study was conducted over approximately 12 months across three phases: (1) theater testing of study procedures, (2) quantitative data and hair sample collection, and (3) qualitative exploration of participant experiences with the hair sampling process.

## METHODS

### Study Setting and Population

The study site is a Women’s HIV Service, located within an academic medical center in the South Florida region of the U.S. Participants were enrolled from various services of the clinic, including prenatal care, primary care, and gynecological specialty care.

### Materials and Content Delivery

Study materials were developed in English and then translated into Spanish and Haitian Creole by certified translators. Study information, written consent materials, hair collection instructions, and interview questions were communicated in the participants’ preferred language in a culturally appropriate manner.

### Eligibility Criteria

1. Cis-gendered women aged ≥18 years;
2. Confirmed HIV diagnosis (as documented in the Women’s HIV Program database)
3. Active patient (i.e., not withdrawn, transferred, or dismissed) receiving care with the Women’s HIV Service
4. At-risk for falling out of HIV care, defined as reporting one of the following:
  - Use of substances including alcohol, marijuana, illicit drugs, non-prescription opioids, and cigarettes
  - Psychiatric diagnosis such as depression, anxiety, or PTSD
  - Currently having sub-optimal HIV care outcomes along the HIV care continuum (i.e. missed visits, not virally suppressed)
5. In any life stage: pregnant, non-pregnant, pre/post-menopausal

#### Exclusion criteria:

1. Not able to provide consent in English, Spanish, or Haitian Creole
2. Cognitively impaired adult (as documented in medical records)
3. Presence of a debilitating psychiatric diagnosis (e.g. schizophrenia/bi-polar/psychotic disorders)
4. Currently incarcerated

### Recruitment Strategy

Participants were recruited and enrolled from a Women’s HIV Service in an academic medical center where approximately 700 unique women are seen annually. The clinic’s census was accessed on a weekly basis to determine eligibility of patients scheduled to be seen the following week. A total of 100 participants were pre-screened before recruitment.

Recruitment for Phase 1 theater testing enrolled the first 2 eligible and interested pre-screened participants from each language group (English, Spanish, and Haitian Creole). Additional participants were recruited as interest allowed, resulting in an uneven distribution across language groups in the final theater testing sample. Phase 2 recruitment then drew from the remaining pre-screened participants who had not been enrolled in Phase 1, ensuring no overlap between the two phases. All pre-screened participants were provided with basic recruitment materials, and interested patients contacted the study team by phone.

### Incentives and Compensation

To compensate for their time, incentives up to a maximum of $80 were offered to participants in the form of cash, electronic transfer, or gift card. Participants received $25 upon enrollment and completion of baseline data collection (approximately 60 minutes) and $25 upon mailing their hair sample in a prepaid envelope. Those who participated in qualitative interviews received an additional $30 for a 75-minute session. All patient data and identifiers for incentive documentation were kept confidential and securely stored.

### Consent to Participate

Written consent was received from all participants in a private room outside the clinic prior to initiating any research procedures. A member of the study staff explained the protocol, reviewed the consent form in full, and clarified questions before obtaining each participant's signature. Participants were given ample time to consider their agreement, and the study team was available to answer any questions about study procedures in their preferred language. If a participant wished to consult with family members before consenting, a follow-up appointment was scheduled to review the consent form again and obtain her signature at that time.

### Data Collection, Management, and Analysis

#### Phase 1: Theater Testing

Phase 1 Theater Testing was conducted to evaluate the feasibility of hair collection as an ARV monitoring method, with feasibility defined as participants' ability to understand and correctly perform the study procedures, rather than their willingness to undergo hair collection or any hesitations about sampling.

Recruited participants were consented and received a slideshow educational session using videos and images covering hair sampling materials, cutting procedures, and mailing instructions. Following the session, participants shared structured feedback on the hair collection process, which was recorded in a spreadsheet to assess their comprehension and ability to execute the required steps for self-sampling.

#### Phase 2: Quantitative Data Collection and Hair Sample Collection and Analysis

Phase 2 combined self-reported health and demographic data collection with hair sampling and education. Self-reported data were collected for future comparison against hair ARV concentrations to identify participant characteristics correlated with adherence patterns. In addition to generating quantitative data, Phase 2 also offered a preliminary indication of the acceptability of the hair sampling procedure, reflected in the proportion of enrolled participants who consented to shipping a hair sample to the laboratory. A more explicit assessment of acceptability was subsequently captured through participant-reported experiences in Phase 3 qualitative data collection.

The team collected self-reported sociodemographic, clinical, and behavioral data from participants in-person or remotely via laptop or tablet at the time of study consent. These data were supplemented by data extracted from the Women's HIV Service database (e.g., HIV/AIDS diagnosis date, viral load, substance use and psychological history, chronic conditions such as hypertension, and gynecological diagnoses such as cervical/anal dysplasia).

Self-reported sociodemographic information (e.g., age, race/ethnicity, place of birth, marital and relationship status, education, employment, income), medication adherence, HIV viral load, CD4 count, and experience with self-collection of biospecimens were collected. Participants also completed seven validated instruments in their preferred language assessing medication adherence [17], internalized HIV stigma [18], medical mistrust of healthcare institutions [19], intersectional discrimination [20], resilience [21], depressive symptoms [22], and drug and alcohol use [23,24]. Item counts, response formats, and Cronbach's α by language are detailed in Table 1. Instruments without prior Spanish or Haitian Creole validation were translated and piloted in previous studies with our research team [25,26], or piloted specifically for this study.

After collecting self-reported data, participants were guided through the hair collection process via the same presentation used for Phase 1 theater testing, which instructed them to cut a small lock of hair close to the scalp, tape the root end, and wrap and seal it in a plastic bag for mailing. Each participant then self-collected their sample at home or at any location outside of the clinic and mailed it to the laboratory for processing. The following figure from the instructional presentation illustrates this process.

#### Phase 3: Qualitative Data Collection

Participants were interviewed to explore their perspectives on the feasibility and acceptability of home-based hair sampling procedures. Data were collected through individual interviews or focus groups of 2 to 8 participants, lasting up to 75 minutes. Sessions were audio-recorded, transcribed, and will be analyzed using password-protected, cloud-based platforms. Focus groups and interviews were facilitated by a study team member in the patients’ preferred language and covered the following topics: overall study experience, likes and dislikes, perceptions, challenges and difficulties, suggested improvements to study instructions, concerns and recommended changes, motivation to participate, and interest in future participation.

### Study Outcomes

#### Phase 1: Theater Testing Outcomes

A total of 5 participants took part in theater testing. While the aim was to recruit 2 participants per language group (English, Haitian Creole, and Spanish), the final sample consisted of 3 English speakers, 1 Haitian Creole speaker, and 1 Spanish speaker, dependent on patient eligibility and interest.

Of the 5 participants, 3 reported hesitation in providing hair samples (2 English speakers and 1 Haitian Creole speaker), while 2 found the process acceptable. The hesitant participants noted discomfort and unwillingness to proceed with hair cutting. However, one participant noted that this type of research could be valuable if it improved how future testing is conducted. Despite the hesitation observed during theater testing, participants demonstrated understanding of the educational content and competence in the hair collection process, meeting the team's definition of feasibility and supporting the decision to proceed to Phase 2.

The contrast between theater testing hesitation and Phase 2 outcomes, where only 3 of 40 recruited participants declined enrollment and 34 of 37 consenting participants ultimately provided a usable hair sample, indicates that hesitancy observed in a small theater-testing sample did not translate into meaningful attrition at scale.

#### Phase 2: Quantitative Sample Collection Outcomes and Data Analysis

Forty (n=40) patients initially agreed to enroll into Phase 2 following theater testing, with 3 withdrawing and 37 consenting to participate in hair sample collection. Two participants subsequently withdrew, resulting in 35 women completing both the educational session and hair sampling process. Of these participants, 60% (n=21) identified English as their primary language, 26% (n=9) identified Spanish, and 14% (n=5) identified Haitian Creole. During the process of mailing samples to the laboratory, 2 samples were lost in transit. Both participants were contacted to resubmit; one agreed to do so while the other declined, resulting in a final total of 34 hair samples received by the laboratory.

Cross-sectional analyses will examine relationships between ARV concentrations in hair samples and self-reported HIV clinical outcomes, including HIV viral load and CD4 count/percentage. Analyses will also assess the extent to which established barriers and facilitators to HIV care adherence like mental health status and substance use, healthcare and supportive service utilization, medical comorbidities, and gynecological diagnoses correlate with HIV-related outcomes.

#### Phase 3: Qualitative Data Analysis

A total of 24 participants from Phase 2 continued to Phase 3 qualitative data collection. Eight participants took part in focus group interviews, conducted across 2 groups of 4 participants each. The remaining 16 participants completed individual in-depth interviews.

To understand barriers and facilitators to hair sampling feasibility and acceptability in this population, qualitative data on participants' lived experiences with the collection process will be analyzed.

Transcript analysis will employ both inductive and deductive approaches, guided by a codebook developed from prior studies. Through iterative review, text segments will be identified, coded, and then grouped into broader themes, with salient themes incorporated into the codebook through ongoing refinement. Frequency and distribution of themes will also be summarized.

### Confidentiality

Confidentiality was maintained by restricting access to IRB-approved personnel. De-identified data were stored in a HIPAA-compliant REDCap database with participant identifiers secured in a separate password-protected file. Audio recordings and qualitative transcripts were de-identified and stored in a secure cloud platform. Data, consent forms, and study logs will be destroyed 3 years after study closure.

#### HUMAN ETHICS AND CONSENT TO PARTICIPATE

This study was approved by the University of Miami Institutional Review Board (IRB #20230023) in January 2023. Procedures were conducted in accordance with institutional and federal ethical guidelines. All participation in the study by subjects was voluntary, with written informed consent obtained from all participants in their preferred language prior to participation.

#### CONSENT TO PUBLISH

Informed consent was obtained from all the individual participants for publication.

#### PRELIMINARY RESULTS

Data from Phases 2 and 3 are currently being analyzed to further evaluate feasibility and acceptability, and to inform future home-based ARV hair collection and testing initiatives. In Phase 2, all 34 hair samples received by the UCSF HAL Laboratory as of March 2026 have undergone baseline evaluation. Of these, 17 require further interpretation pending clinical correlation. In Phase 3, data analysis is ongoing for 2 focus group (n=8) and n=16 individual interview transcripts.

#### FUNDING

This study was funded by the National Institutes of Health (NIH) through the National Institute of Drug Abuse (NIDA) grant R25DA028567.

## DISCUSSION

This protocol is designed to evaluate the feasibility and acceptability of home-based hair sample collection for ARV drug level testing among a racially, ethnically, and linguistically diverse cohort of clinic-based women with HIV in South Florida who are at risk of falling out of care. Preliminary results support the feasibility and acceptability of this methodology. Theater testing demonstrated feasibility through understanding and appropriate performance of sampling procedures, while Phase 2 data collection demonstrated acceptability as hair samples were provided by 35 out of 40 women, with 34 successfully reaching the laboratory for further processing.

These findings are particularly meaningful given that the study population is underrepresented in HIV-related adherence research, and women of color experience disproportionate structural and individual-level barriers to HIV care and viral suppression [28,29]. The linguistic composition of our study reflects a more diverse sample than prior hair-based ARV adherence research, which was conducted exclusively in English among a predominantly White, male cohort [13,14]. Home-based ARV analysis may therefore offer a low-barrier method of monitoring drug levels for linguistically diverse women.

### Strengths

Despite initial hesitation reported during theater testing, refusal rates were ultimately low, suggesting that concerns surrounding hair collection may be mitigated through culturally sensitive communication, self-collection techniques, and intrinsic motivation.

Prior literature identified cultural and religious significance related to hair in areas such as West Africa, India, and the Caribbean, which impacted hair sampling [30-32]. In Nigeria and Kenya, individuals have declined to donate hair for biomedical research over concern that samples could be diverted for ritual use [33-35]. Among women with HIV in South Africa, hesitation about providing hair samples for ART adherence monitoring stemmed from potential conflict with their own spiritual beliefs or those of others [36]. This is particularly relevant to our study population, as some Caribbean and Latine cultures similarly attach strong identity and spirituality to hair and bodily materials [37,38]. However, these concerns did not substantially limit participation in the present study.

High participation may reflect the study team’s emphasis on clear communication with participants (a presentation providing step-by-step directions delivered in participants' preferred language), participant-directed home-based collection, and direct mailing of samples to the laboratory. Prior studies have found that clear communication, respecting hair's cultural meaning, self-collection at home, and gentler cutting techniques can improve trust and acceptability of hair collection [39].

Additionally, some participants in this study had prior experience in biomedical research, which may have familiarized them with research procedures and sample collection processes, a factor previously linked to willingness to donate hair for research [34].

While not formally quantified, interviewers noted that many participants, particularly those living long-term with HIV, expressed a strong altruistic motivation to contribute to research that could benefit other women in the future, which appeared to outweigh potential hesitation related to hair collection. Similar motivations were observed across HIV studies globally, with participants reporting that the opportunity to help advance HIV treatment drove their willingness to provide biospecimens [40].

### Limitations

Several limitations should be considered. Selection bias may have occurred, as participants were recruited during clinic visits and self-initiated contact with study personnel. Although these participants are still at risk of falling out of care, their engagement at enrollment suggests their access to care may distinguish them from the broader population of women with HIV at risk of falling out of care, potentially inflating study acceptability.

Additionally, cosmetic hair treatments may affect ARV detection and introduce variability in drug concentrations. Studies found that certain antivirals may not be detectable following dye, bleach, or relaxer treatment, which may restrict the eligible sample population in future studies and could explain why further interpretation and analysis were needed for some of the samples collected in this study [41,42].

Feasibility was also constrained by logistical challenges in the collection and mailing process. Two samples were lost in transit, with one participant declining to repeat collection after sample loss, highlighting the importance of reliable tracking systems and minimizing participant burden in future studies.

## Conclusion and Future Directions

Preliminary findings suggest that home-based hair sample collection is a feasible and acceptable approach for ARV monitoring among diverse, multilingual women with HIV who are at risk for disengagement from care. As a non-invasive measure of cumulative ARV exposure, hair-based analysis may complement existing measurement and adherence assessment strategies, particularly for individuals facing barriers to routine clinic attendance and laboratory test analysis. Findings to-date indicate that clear education combined with culturally responsive, self-directed collection procedures outweighed hesitation around biological sampling reported in prior literature, particularly among women with long-term HIV diagnoses motivated to contribute to research benefiting others.

Future studies should continue to prioritize culturally sensitive communication and further evaluate the effects of cosmetic hair treatments on ARV detection. Additional research is also needed to assess the integration of hair-based adherence monitoring into routine HIV care and its impact on long-term adherence, viral suppression, and retention in care.

## Figures and Tables

**Figure 1:**
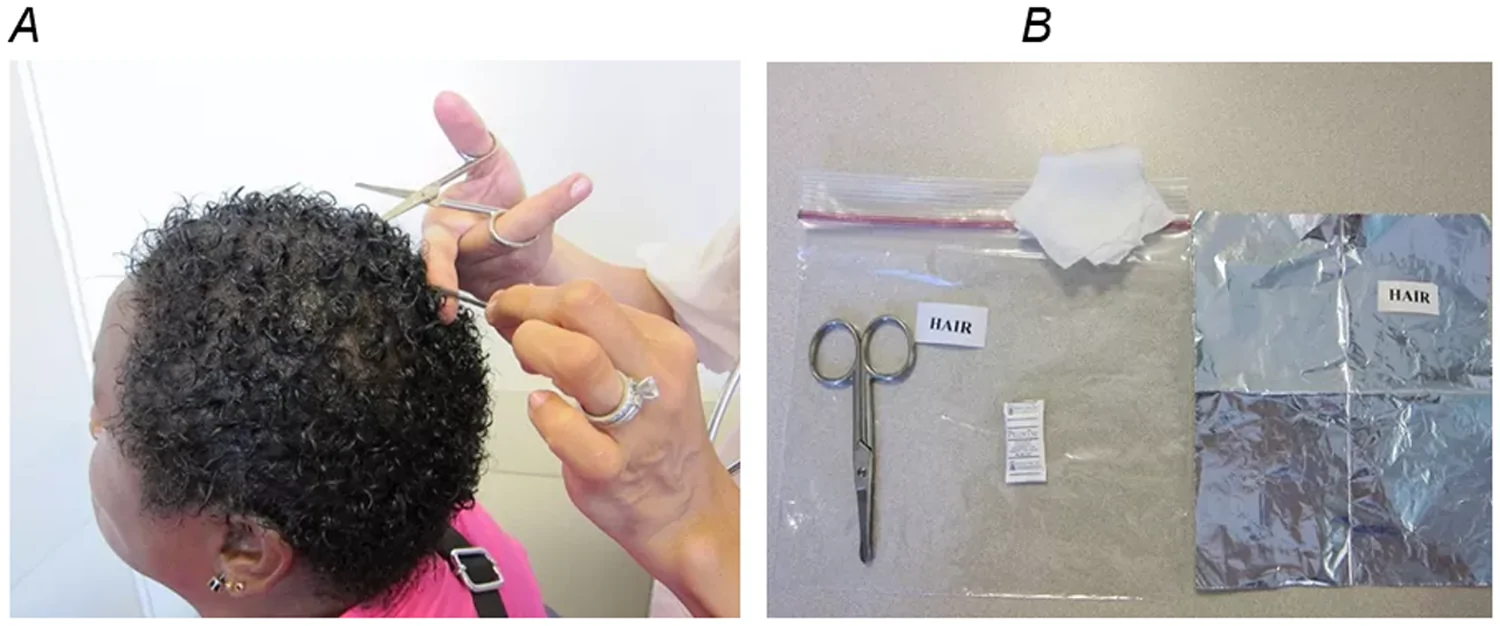
**1a and Fig. 1b** Images used in educational presentation to demonstrate hair sampling process [27] Samples are currently being analyzed for ARV levels. Results have not yet been reported and were collected for research purposes only.

**Figure 2:**
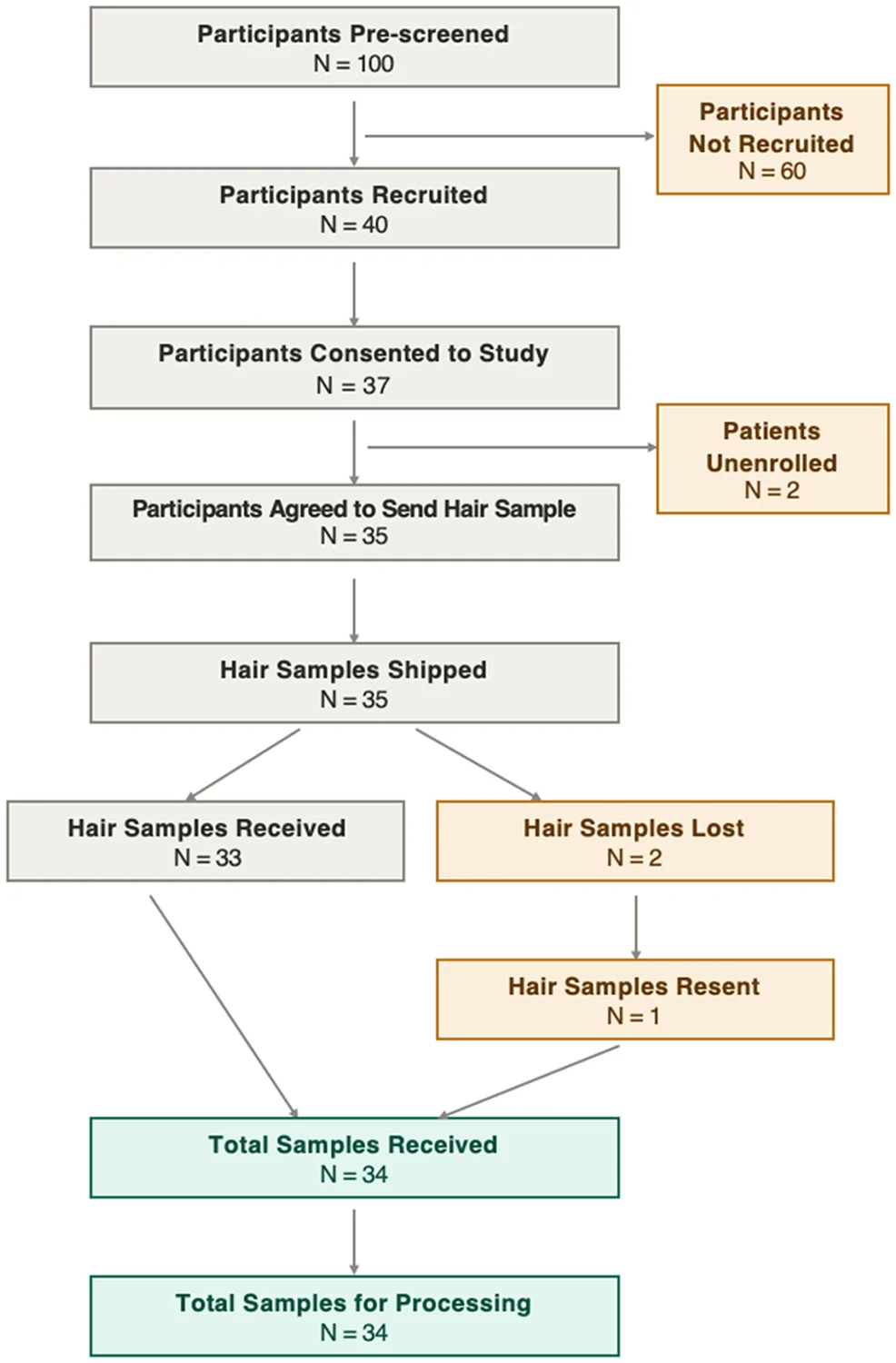
Participant flow through recruitment, enrollment, hair sample collection, and sample preparation for processing

**Table 1.** Data instruments used for self-reported information

Instrument Name	Measures	# of Items	Response Scale	Validated English Cronbach's α	Validated Spanish Cronbach's α	Validated Creole Cronbach's α
**Demographics, Financial Information, Viral Suppression & Biospecimen Experience (Self-Reported)**	Sociodemographic info (age, race/ethnicity, birthplace, marital/relationship status, education, employment, income), medication adherence, HIV viral load, CD4 count, and experience with self-collection of biospecimens	14	Mixed (categorical, numeric, yes/no)	Piloted with this study	Translated and piloted with this study	Translated and piloted with this study
**Three-Item Self-Report Measure for Medication Adherence** [17]	Medication adherence (days taken, frequency, and rating of medications over the past 30 days)	3	Likert-type scale	Validated α = .84	Translated and piloted with prior study [25]	Translated and piloted with prior study [25]
**HIV Stigma Scale** [18]	Internalized stigma regarding HIV	40	Likert-type scale	Validated α = .96	Validated α = .91	Translated and piloted with prior study [25]
**Group-Based Medical Mistrust Scale** [19]	Medical mistrust against doctors and healthcare institutions	12	Likert-type scale	Validated α = .64–.70	Validated α = .80	Translated and piloted with prior study [26]
**Intersectional Discrimination Index (InDI)** [20] (InDI-A: 9 items, InDI-D: 9 items, InDI-M: 13 items)	Discrimination across multiple intersecting identities (anticipated, day-to-day, and major discrimination)	31 (3 subscales)	Likert-type scale	Validated α = .93	Translated and piloted with this study	Translated and piloted with this study
**Connor-Davidson Resilience Scale (CD-RISC-25)** [21]	Resilience	25	Likert-type scale	Validated α = .89	Validated α = .89	Validated α = .80
**Patient Health Questionnaire-9 (PHQ-9)** (22)	Depressive symptoms over the past two weeks	9	Likert-type scale	Validated α = .89	Validated α > .89	Validated α = .78
**Drug Abuse Screening Test-10 (DAST-10)** [23]	Drug use behaviors and consequences in the past 12 months	10	Binary (Yes / No)	Validated α = .86–.94	Validated α = .89	Translated and piloted
**Alcohol Use Disorders Identification Test-Concise (AUDIT-C)** [24]	Frequency and quantity of alcohol consumption; risk for hazardous use	3	Likert-type scale	Validated α = .80–.95	Validated α = .88	Translated and piloted with this study

## Data Availability

The data analyzed during this study contain sensitive health information, including HIV status, and are protected under a Certificate of Confidentiality issued by the NIH. These data are not yet publicly available, as analysis is ongoing. In line with institutional policy, de-identified data may be made available from the corresponding author upon reasonable request after completion of the study.
